# Hybrid CNN–Transformer Network for Electricity Theft Detection in Smart Grids

**DOI:** 10.3390/s23208405

**Published:** 2023-10-12

**Authors:** Yu Bai, Haitong Sun, Lili Zhang, Haoqi Wu

**Affiliations:** School of Electronical and Information Engineering, Shenyang Aerospace University, Shenyang 110136, China; yubai@sau.edu.cn (Y.B.); 20052727@sau.edu.cn (L.Z.); wuhaoqi@stu.sau.edu.cn (H.W.)

**Keywords:** electricity theft detection, transformer neural network, convolutional neural network, smart grids

## Abstract

Illicitly obtaining electricity, commonly referred to as electricity theft, is a prominent contributor to power loss. In recent years, there has been growing recognition of the significance of neural network models in electrical theft detection (ETD). Nevertheless, the existing approaches have a restricted capacity to acquire profound characteristics, posing a persistent challenge in reliably and effectively detecting anomalies in power consumption data. Hence, the present study puts forth a hybrid model that amalgamates a convolutional neural network (CNN) and a transformer network as a means to tackle this concern. The CNN model with a dual-scale dual-branch (DSDB) structure incorporates inter- and intra-periodic convolutional blocks to conduct shallow feature extraction of sequences from varying dimensions. This enables the model to capture multi-scale features in a local-to-global fashion. The transformer module with Gaussian weighting (GWT) effectively captures the overall temporal dependencies present in the electricity consumption data, enabling the extraction of sequence features at a deep level. Numerous studies have demonstrated that the proposed method exhibits enhanced efficiency in feature extraction, yielding high F1 scores and AUC values, while also exhibiting notable robustness.

## 1. Introduction

With the advancement of smart grid technology and the ongoing expansion of power system infrastructure, the power industry, as a fundamental sector facilitating national economic growth, has increasingly emphasized the need to enhance the economic efficiency and ensure the stable operation of power companies [1]. The categorization of electricity losses can be divided into two main types: technical losses (TLs) and non-technical losses (NTLs) [2]. Technical losses are a result of disparities in infrastructure and energy dissipation, whereas non-technical losses emerge from the disparity between the total power transferred over distribution lines and the power consumed by customers. Electricity theft is the predominant type of non-technical loss, encompassing a range of techniques including private cables, physical manipulation of meter counting components, and destructive modification of meter facilities resulting in inconsistent meter readings [3]. Electricity theft not only carries significant economic consequences for the nation but also poses a threat to public safety, since it heightens the risk of mishaps such as fires and electric shocks. According to the source cited as [4], the aggregate financial impact of power theft on a global scale is estimated to be around CAD 100 million per year. This substantial amount of money, if not lost to theft, might instead be utilized to supply electricity to around 77,000 households for a duration of 1 year.

Numerous potential resolutions to the issue of power theft have been put out in the existing body of scholarly work [5,6,7]. The existing body of literature classifies these solutions into two primary categories: hardware-based solutions and data-driven solutions. Hardware-based solutions primarily center around the development of intelligent devices and sensors with the capability to identify and detect irregularities. Nevertheless, it should be noted that the aforementioned solutions incur significant maintenance expenses, exhibit lower levels of efficiency, and require a substantial amount of time [8]. Furthermore, they demonstrate an elevated false-positive rate (FPR). On the other hand, there exists a plethora of data-driven methodologies aimed at detecting instances of electricity theft [9]. These solutions utilize methodologies rooted in artificial intelligence (AI) [10], game theory (GT) [11], and machine learning (ML), which are extensively applied in various fields such as healthcare, education, and transportation. According to cited source [12], solutions that are driven by data have enhanced resilience, efficiency, and comprehensibility. Furthermore, the scholarly literature [13] presents a methodology centered on grid analysis as a means to examine the identification of abnormal power consumption patterns. This methodology involves scrutinizing several parameters of the grid, such as current, voltage, and others, in order to find any atypical usage behavior. Anomaly detection encompasses the utilization of diverse data types, encompassing network-related data such as the operational state of switches and circuit breakers, alongside sensor data like voltage and current magnitudes captured by remote terminal units.

During the early phases of classification research, conventional machine learning techniques [14,15] were employed for the purposes of feature extraction and classification. The approaches employed in this study encompassed support vector machines (SVMs) [16,17], decision trees (DTs) [18,19], and nearest neighbors [20,21]. With the advancement of machine learning algorithms, there has been an increasing adoption of integrated learning algorithms that consist of several individual learners for the purpose of power theft detection. Several studies have presented several strategies for detecting instances of electricity theft, utilizing integrated learning algorithms such as random forest (RF), Adaboost, and XGBoost [22,23,24,25]. The experimental findings provided evidence that the integrated learning algorithms exhibit superior performance compared to conventional approaches. In a specific research investigation [26], deep learning models [27] were utilized as binary classifiers, with the purpose of detecting instances of energy theft. The researchers examined various deep learning architectures, such as CNN, Multi-Layer Perceptron (MLP), Long–Short-Term Memory (LSTM), and Gated Recurrent Unit (GRU) networks. Pereira et al. [28] employed a CNN for the purpose of detecting instances of power theft. Additionally, they conducted a comparative analysis of different oversampling approaches to investigate the potential effects of dataset imbalance. Zheng et al. [29] utilized a CNN to extract periodic features from load data that were transformed into a two-dimensional format. These extracted features were subsequently combined with global characteristics acquired from one-dimensional load data, which were captured using a fully connected network. The purpose of this approach was to detect instances of power theft. In a separate investigation, the authors of study [30] employed a fusion of clustering algorithms and Long–Short-Term Memory networks in order to identify instances of electricity theft. The methodology employed entailed forecasting the subsequent electricity usage of a client at each given time and afterwards evaluating the disparity between the projected values and the actual data. Deep learning techniques provide the advantage of automated sequence feature extraction in comparison to conventional machine learning algorithms.

The issue of detecting electricity theft has been extensively explored in academic research, leading to the development and widespread adoption of several hybrid neural network models that incorporate deep learning techniques. The study conducted by [2] introduced a hybrid neural network that integrates Long–Short-Term Memory (LSTM) and Multilayer Perceptron (MLP) models. This hybrid network demonstrates the ability to extract characteristics from diverse data sources. The authors Ismail et al. [31] proposed a hybrid neural network model that combines CNN and GRU to tackle the issue of electricity theft in distributed generation systems. The researchers in [32] devised a novel hybrid neural network architecture that integrates the GRU, CNN, and Particle Swarm Optimization (PSO) algorithms. This model was trained and evaluated using real-time data on electricity use. The utilization of the CNN facilitates the reduction of dimensionality and redundancy within time series data. The classification of consumption patterns into normal and fraudulent categories is achieved by the utilization of the GRU network and particle swarm algorithm. The integration of the long- and short-term memory strategies into CNN technology was found to boost e-fraud detection, as demonstrated in a study conducted by [33]. The optimal values of the hyperparameters for the CNN–LSTM were computed using meta-heuristic techniques, namely Black-Widow Optimization (BWO) and Blue-Monkey Optimization (BMO). The aforementioned works [30,31,32,33] have introduced detectors that function as hybrid deep-learning models, specifically designed for the purpose of feature extraction.

The aforementioned models have demonstrated favorable outcomes in the domain of electricity theft detection, yet certain concerns persist. The initial approach in many electrical theft detection models relies on CNNs. However, CNNs have limitations in properly capturing the global characteristics of time series data and calculating the relative correlations among the retrieved features. The excessive dependence on the initial input data presents a notable limitation. Furthermore, it is possible for the model to experience overfitting as a result of the disparity between the amount of data available in the training set and the intricacy of the model. As a result, the model’s capacity to generalize to real-world scenarios is constrained. Therefore, it is imperative to consider the importance of mitigating model overfitting and improving feature extraction capabilities. Ding et al. [34] introduced a multivariate-branching block (DBB) as a means to extract feature information. The DBB accomplishes this by integrating several branches with diverse widths and complexities. The Gaussian-weighted feature-tokenization transformer module (FTT) was introduced by Sun et al. [35]. The FTT module aims to investigate the transformer’s ability to capture local spatial semantic information and effectively represents the links between adjacent sequences. Moreover, Shi et al. [36] introduced a novel methodology for detecting power theft through an end-to-end approach by utilizing the transformer neural network. This study presents a novel hybrid model named the DSDB CNN and the Gaussian-weighted transformer network (DSDBGWT), which integrates a CNN with a DSDB structure and a GWT network. In contrast to a CNN, the DSDBGWT model demonstrates enhanced proficiency in extracting global features and determining the relative relationships among various characteristics. As a result, it diminishes its dependence on the initial input data when performing classification tasks. In order to augment the model’s ability to extract features, a GWT module is utilized, which is particularly well-suited for processing sequences of extended duration. The present module effectively captures the characteristics of extended temporal sequences through the computation of attention coefficients, which are determined by the positional information of the input sequences. As a result, the model demonstrates enhanced efficacy in the detection of electricity theft. In order to address the issue of overfitting in the model, the initial step involves incorporating suitable normalization layers (LN) into both the regular block and transformer block. Furthermore, the dropout regularization technique is utilized to stochastically deactivate a certain proportion of neurons throughout the training process.

The main contributions of this article are summarized as follows:

(1) We propose a simple and efficient DSDB convolutional module in our network to extract inter- and intra-periodic features from sequences. This module replaces the traditional CNN structure, resulting in a lightweight model while improving model accuracy;

(2) We employ a transformer network with Gaussian weighting. The attention weights in this network can be attenuated based on the distance between related symbols. This allows for a more rational allocation of the attention mechanism, leading to more efficient extraction of sequence features and improved model accuracy;

(3) The systematic combination of CNN network and GWT network can fully extract the electricity consumption information in the sequences and accurately and efficiently recognize the semantic features, thus significantly improving the classification accuracy. Extensive experiments on the China National Grid dataset show that our DSDBGWT model outperforms other existing methods.

The remainder of this paper is structured as follows. Section 2 presents the framework of the proposed model and provides specific details on the implementation of its constituent modules. In Section 3, we elaborate on the dataset processing, conduct comparative experiments to assess the effectiveness of our framework, and discuss the experimental results. Finally, Section 4 concludes the paper.

## 2. Materials and Methods

**Figure 1:**
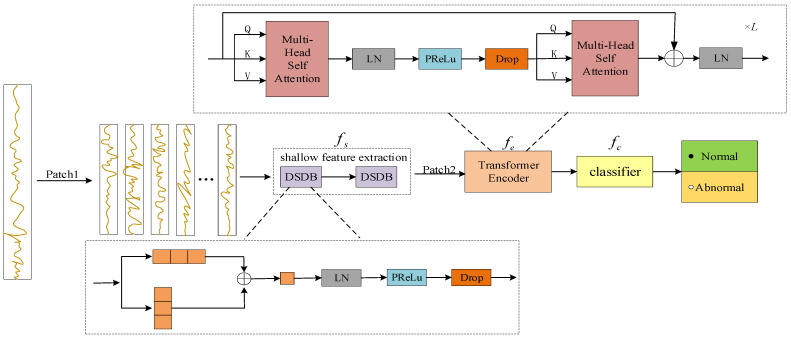
The overall architecture of the proposed hybrid neural network, combining DSDB convolutional neural network and GWT network (DSDBGWT).

The overall architecture of the hybrid model for electricity theft detection (DSDBGWT) based on a CNN with DSDB and a GWT network is shown in Figure 1. The framework has three distinct modules: a CNN that incorporates a DSDB structure to facilitate shallow feature extraction, a GWT network designed specifically for long-distance feature extraction, and a classification module. Initially, the original sequence is segmented on a weekly basis using patch [37] to effectively capture the overall characteristics and minimize computing workload, while still retaining the information from the original sequence. Following the implementation of the patch, two DSDB structures are employed, possessing identical structures. This approach enables the extraction of inter- and intra-week features of electricity consumption information with enhanced accuracy and efficiency. Additionally, this significantly reduces the computational burden associated with the convolutional operation. Subsequently, the output data generated by the CNN are once again divided into discrete four-week intervals, employing patches as the input for the transformer model. The GWT network is capable of extracting a sequence’s global features, which can produce varying weighting weights based on the input data’s distance. Thus, enhancement in feature extraction accuracy is achieved. It is important to highlight that this approach differs from the standard transformer in that it does not incorporate a class token and position embedding into the transformer’s tokens. Consequently, it does not engage in MLP processing within the tokens, but instead prioritizes the extraction of deep features from the tokens. Ultimately, the outcomes of the encoding process for each token are fed into the classification module.

The DSDBGWT network model proposed in this paper can be denoted as $f=f_{c}⊙f_{e}⊙f_{s}$, with parameters $\omega =\{\omega_{s},\omega_{e},\omega_{c}\}$. Here, $f_{s}$ is a convolutional neural network, used for shallow feature extraction, and its output is $v=f_{e}(x;\omega_{e})$; $f_{e}$ is a transformer network, used for long-distance feature extraction, and its output is $z=f_{e}[f_{s}(x;\omega_{s});\omega_{e}]$; and $f_{c}$ is a classifier, used for result categorization, which maps instances from the representations to the corresponding logics, which can be transformed into similar classes by $p(y|z;\omega )=sigmoid(f_{c}(z;\omega_{c}))$. We optimize the end-to-end parameters by minimizing the cross-entropy loss on the set of markers denoted as $\sum_{(x,y)∼D_{train}}[l(f_{c}⊙f_{e}⊙f_{s}(x;\omega ),y)]$. We define x to represent the input data, p(;) to represent the derived probability value, and $\sum_{\left(x,y\right)}$ to represent the sum of the loss functions.

### 2.1. Data Preprocessing

**Figure 2:**
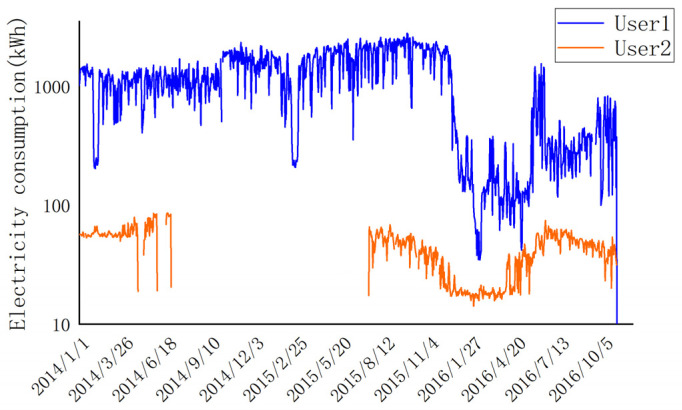
Display of electricity consumption by the largest user in the dataset and by an average user.

The suggested approach is used for the smart meter data of consumers’ daily electricity consumption, which is sourced from the State Grid Corporation of China (SGCC) [38]. The dataset provided includes authentic power consumers as well as those engaged in electricity theft, with more information about the dataset available in Table 1. Figure 2 illustrates the electricity consumption patterns of two users within the dataset. User 1 exhibits the highest electricity usage, with a daily consumption reaching close to 2000. In contrast, User 2 represents the majority of electricity users, ranging from a few kWh to a dozen kWh per day. This discrepancy highlights the significant variation in electricity consumption among users. To address this, it is necessary to normalize the data. Normalization not only stabilizes the dataset but also enhances the convergence speed and overall efficiency of the model. Furthermore, it is evident that the data from User 2 exhibits discontinuity in certain instances. This can be attributed to various intricate factors encountered during the meter collection process, such as unreliable transmission of data due to smart meter faults, irregular system maintenance, occurrence of special events, and other multifaceted elements. Consequently, these factors contribute to the absence of electricity consumption data. In order to mitigate the impact of data variations on the neural network model, it is imperative to employ appropriate data preprocessing techniques. This study undertakes the normalization of raw data and addresses the issue of missing values through appropriate processing techniques.

(1) The process of normalization.

The act of normalizing the dataset has the effect of increasing the numerical conditions of the dataset, which in turn enhances the stability of the optimization method. Consequently, this phenomenon enhances the speed of model training and augments the efficiency of the algorithm. In addition, the process of normalization serves to standardize the distribution of data and reduce the influence of outliers on the model, improving its resilience. We choose the scaling method of MAX−MIN to normalize the data according to the following equation. In the normalization process, we leave the missing values untouched first:

(1) $$n(x)=\frac{x-\min(x)}{\max(x)-\min(x)}$$

Here, x represents the user’s electricity consumption on a specific day, while min⁡(x) and max⁡(x) represent the minimum and maximum values, respectively, across the entire dataset.

(2) Missing value processing.

Missing values are predominantly observed when there is a lack of data at a particular point in time, typically resulting from mistakes in the measuring instrument. The inclusion of these omitted values serves to improve the overall quality of the data, enhancing its trustworthiness and suitability for analytical and modeling purposes. The zero-replacement approach is employed to address the presence of missing data that meet the specified requirements:

(2) $$f(x_{t})=\left\{\begin{array}{l} 0x_{t}\in NAN\\ x_{t}x_{t}\notin NAN \end{array}\right.$$

where $x_{t}$ indicates the user’s electricity consumption at a given time and $x_{t}\in NAN$ indicates that $x_{t}$ is a null value.

The network encountered difficulty distinguishing between the original value being zero and the missing value being imputed as zero, due to the preexistence of zero values in the samples. In order to tackle this matter, we implemented an additional input channel by using a binary mask [39]. Within the mask matrix, the original data’s missing value is designated as 0, whereas the normal value of 0 is designated as 1. By employing this approach, the neural network is capable of differentiating between these two situations, thereby improving the resilience of the model.

The initial dataset, denoted as X, comprises the electricity consumption data for a specific electricity user (referred to as M) over a time period of L days in the past. Therefore, we can represent the original dataset as $X\in R^{M\times L}$. The dataset undergoes preprocessing, which involves normalizing the raw data, processing missing values, and adding binary masks. These processes transform the dataset from a two-dimensional structure to a three-dimensional structure for variable $X^{\prime }\in R^{M\times L\times 2}$.

### 2.2. Patch

Due to the considerable length of the sample sequence, it is necessary to employ the patch technique to partition the data into several subsequences at specific intervals. This strategy not only maintains the intrinsic properties of sequence but also enables more effective management and processing of the data for a range of activities, such as model training, feature extraction, and predictive analytics. The length of the patch is represented by the variable P. The sampling step is marked as S. The total number of patches is indicated by the variable N. The electricity usage per user over a period of L days is symbolized by the variable L. The calculation formula can be expressed as follows:

(3) $$N=\left\lfloor \frac{\left(L-P\right)}{S}\right\rfloor +1$$

**Figure 3:**
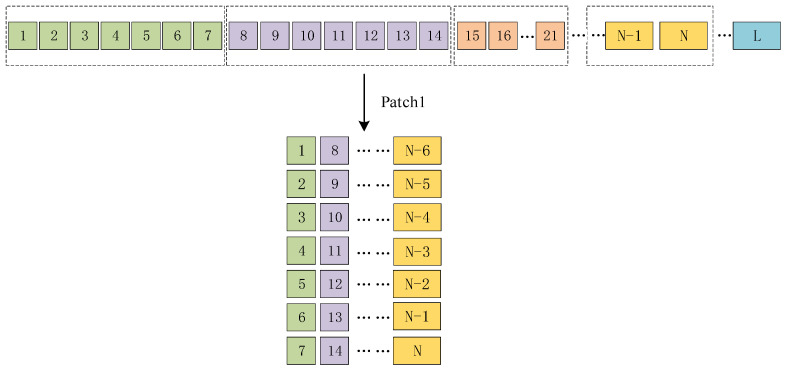
Patch architecture for CNN (N followed by data that are not divisible).

**Figure 4:**
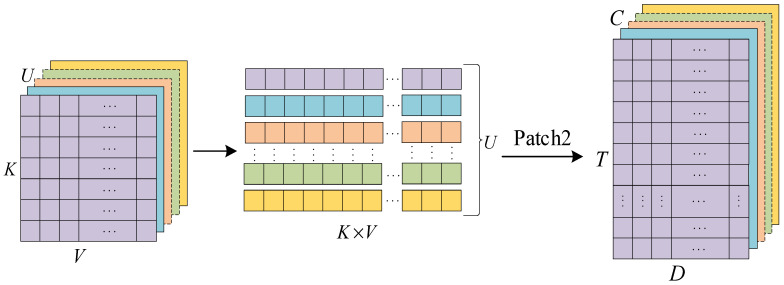
Patch architecture for transformer networks (each channel is patched separately).

Electricity consumption data for normal users are usually more cyclical than for abnormal users [29,40,41] (detailed analysis in Section 3.3). To effectively process such periodic data using CNN, we employ the Patch architecture. Taking a specific sample as an example, the preprocessed data has a spatial size of H×W. Utilizing the Patch architecture and considering the weekly periodicity of the data, we set the parameters P = 7 and S = 7, thus transforming the data into a three-dimensional space represented as $H\times (\left\lfloor \frac{\left(L-P\right)}{S}\right\rfloor +1)\times S$ after Patch processing, as illustrated in Figure 3. Similarly, as shown in Figure 4, the inputs to the transformer network undergo processing using Patch. The decision to employ Patch processing on a four-week cycle is motivated by the transformer network’s exceptional feature extraction capabilities and its proficiency in capturing distant features. The parameters Patch_size = 28 and Stride_size = 28 were set to partition the data based on monthly time intervals. This Patch architecture transforms the dimensionality of the output data from U×K×V to U×(K×V), then to $U\times (\left\lfloor (\frac{K\times V-P)}{S}\right\rfloor +1)\times S$. Moreover, the Patch operation reduces the number of input channels from L to approximately $\frac{L}{S}$, resulting in a reduction in computational complexity by a factor of S. Additionally, the Patch operation enables the model to have a stronger ability to refer back to earlier data, enhancing the network’s learning capability and leading to significant improvements in prediction performance.

### 2.3. Shallow Feature Extraction for DSDB Structures

**Figure 5:**
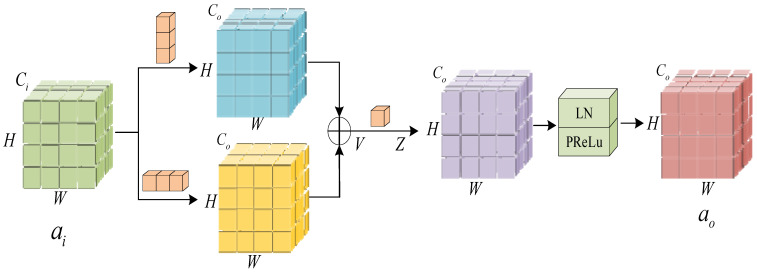
Convolutional neural network model with DSDB structure.

After performing data preprocessing, the sequence features of the samples are extracted using 2D convolution. Figure 5 depicts the implementation of a DSDB structure during this phase. Each branch within the structure incorporates a convolution kernel of different scales, enabling the extraction of more complete feature information compared to a single convolutional network. Taking inspiration from work with ACNet [42], we propose the incorporation of asymmetric convolution into our approach. Specifically, we construct the convolution kernels for each branch to have dimensions of 1 × s and s × 1, respectively. The convolution kernel with dimensions s × 1 is utilized for feature extraction within a singular cycle, whereas the 1 × s convolution kernel is employed for extracting features across different cycles. The process involves the linear combination of two convolutions that are applied to the same locations but on different channels. This results in the emphasis of the squared convolution kernel in both horizontal and vertical directions, thereby highlighting distinct locally prominent features from various orientations. After the integration of the outputs from both branches into a single DSDB output, a 1 × 1 convolutional kernel is employed to maintain the inherent structure of the original sequence. In order to accelerate the rate at which the model converges during training and improve the overall generalization ability of the network [43], a normalization layer is implemented following the convolutional layer in each branch. The normalization layer is responsible for ensuring the normalization of the feature mapping in each branch. This process results in the output features becoming nonlinear and effectively reduces data dispersion. Additionally, it serves as a preventive measure against problems such as gradient explosion or gradient vanishing. Following the normalizing procedure, the PReLu activation function is employed to counteract linearity inside the network, so enabling the network to acquire knowledge about nonlinear mappings in a hierarchical fashion. Ultimately, the utilization of Dropout serves as a means to change data in order to mitigate the occurrence of overfitting.

The model employs two distinct DSDB structures, and the subsequent description pertains to both of these DSDBs. The input data of the DSDB CNN are denoted as $a_{i}\in R^{H\times W\times C_{i}}$, where H×W represents the spatial size and $C_{i}$ represents the number of input channels. The mathematical expression for the DSDB convolution at point (h,w) of its jth channel can be represented by the following formula:

(4) $$V_{j}^{h,w}=\sum_{k}^{C_{i}}\sum_{h^{\prime }=0}^{S}\omega_{v_{1},j,k}^{h^{\prime },0}\cdot a_{i,k}^{h+h^{\prime }-\left\lfloor \frac{S}{2}\right\rfloor ,w}+\sum_{k}^{C_{i}}\sum_{w^{\prime }=0}^{S}\omega_{v_{2},j,k}^{0,w^{\prime }}\cdot a_{i,k}^{h,w+w^{\prime }-\left\lfloor \frac{S}{2}\right\rfloor }$$

Here, $v_{1}$ represents the s × 1 convolution kernel, $v_{2}$ represents the 1 × s convolution kernel, k represents the sum of $C_{i}$ channels, $h^{\prime }$ represents the corresponding position ranging from 1 to s in the s × 1 convolution kernel, and, similarly, $w^{\prime }$ represents the corresponding position ranging from 1 to s in the 1 × s convolution kernel. To maximize data utilization, it is essential to employ the padding operation by adding zeros around the space H×W. The padding size is determined by $\left\lfloor \frac{S}{2}\right\rfloor$; at this point, $V\in R^{H\times W\times C_{O}}$.

Following the DSDB structure, a 1 × 1 convolution kernel is utilized to perform the convolution operation. Consequently, the value at the (h,w) position of the jth channel can be obtained as follows:

(5) $$Z_{j}^{h,w}=\sum_{l}^{C_{o}}\omega_{z,j,l}\cdot V_{l}^{h,w}$$

where l represents the sum of $C_{O}$ channels. At this point, $Z\in R^{H\times W\times C_{O}}$.

Finally, we perform linear normalization processing and use PReLu activation function to obtain $a_{o}=\phi (\gamma Z+\beta )$.

### 2.4. Gaussian-Weighted Transformer Encoder Module

Regarding the detection of electricity theft, past research has predominantly concentrated on shallow feature extraction using convolutional neural networks, resulting in favorable outcomes. However, when addressing the issue of electricity theft, it is crucial to consider the correlation of data over an extended period. The data samples in this case consist of long time series. CNN has limitations in representing features for such long-time series data. The shallow feature extraction of CNN restricts their ability to capture long-term dependencies, as the extensive use of convolutional operations can only encompass a limited range of features. Moreover, the sample data contain a small number of anomalous samples, accounting for only 8.5% of the total. Relying solely on CNNs not only fails to extract more positive outcomes, but also runs the risk of gradient vanishing. To address the challenge at hand, this study introduces a transformer network into the framework. By incorporating the transformer network, the model is able to effectively capture global dependencies, enabling the extraction of long-distance characteristics. Additionally, the transformer network offers parallel computing capabilities, enhancing the overall efficiency of the network. This paper aims to enhance the precision of the model by enhancing the transformer network’s Gaussian-weighted attention mechanism. The proposed improvement involves incorporating a Gaussian-weighted self-attention mechanism into the original network. This mechanism combines features extracted from $W^{Q}$, $W^{K}$, and $W^{V}$ using a Gaussian-weighted matrix, thereby eliminating the reliance on attention weights for feature utilization. The weights undergo attenuation based on the proximity of tokens, with the degree of attenuation being defined by the Gaussian variance. This variance is acquired through the training process. The proposed method has the capability to comprehensively and precisely capture the temporal dependencies on a worldwide scale inside electricity consumption data. Consequently, this approach has the potential to enhance the effectiveness of power theft detection to a greater extent.

**Figure 6:**
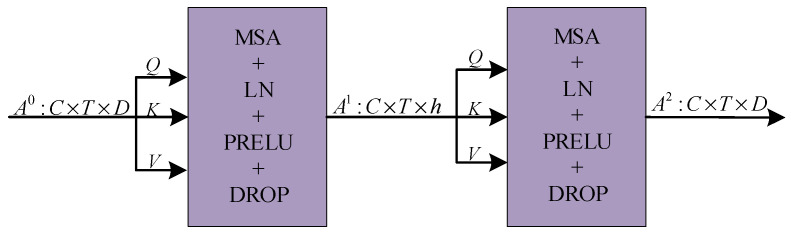
The matrix dimension of input and output features.

**Figure 7:**
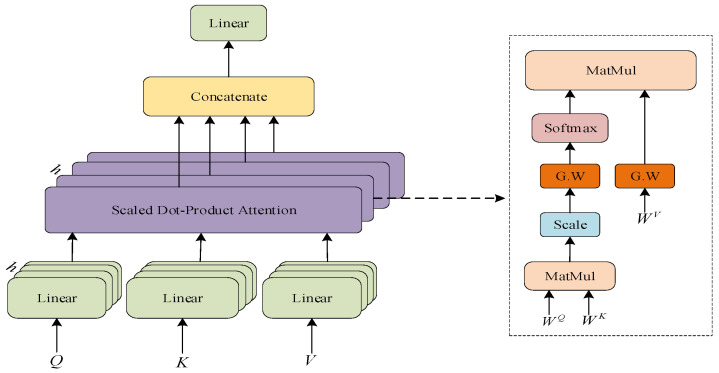
Multi-head self-attention module and the internal structure of a single self-attention.

The module comprises two blocks of multi-head self-attention mechanism (MSA), as depicted in Figure 1. The residual operation is iterated by using the input channels as the heads of the first MSA, and mapping the output of the first MSA to the second MSA as the input heads of the second MSA, with the same dimensions for $A^{0}$ and $A^{2}$. The matrix dimensions of the input and output features are shown in Figure 6. To encompass the global relationship, the multi-head attention mechanism incorporates three learnable weight matrices, namely, $W^{Q}$, $W^{K}$, and $W^{V}$. The ith self-attention (SA) is chosen using the three learnable weight matrices mentioned above. It is then linearly normalized to obtain Scaled Dot-Product Attention, as depicted in Figure 7. This section introduces the concept of Gaussian-weighted self-attention, which allows for the utilization of varying weights based on the proximity of tokens. This feature enhances the accuracy of the findings obtained.

The formula for the SA mechanism is as follows:

(6) $$SA=Attention(Q,K,V)=soft\max(\frac{QK^{T}}{\sqrt{d_{K}}})V$$

where $d_{K}$ is the dimension of K.

**Figure 8:**
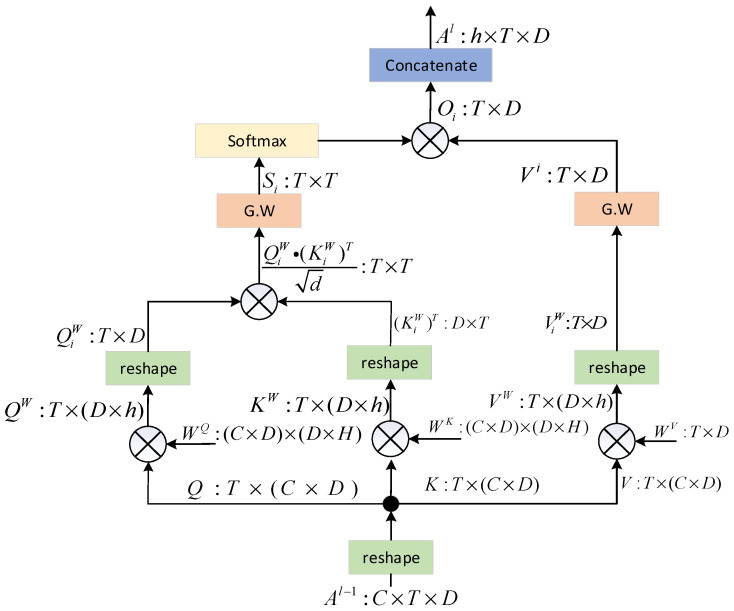
Proposed multi-head self-attention block diagram. The G.W. block performs element-wise multiplication of the Gaussian-weight matrix with the generated score matrix. The matrix dimensions are noted beside each signal.

The diagram illustrating the internal architecture of Gaussian-weighted self-attention is depicted in Figure 8. In this context, B represents the size of the batch, *T* defines the length of the sequence, D marks the dimension of the input, and E relates to the number of units in the self-attention mechanism. The matrices for the query, key, and value are defined in the following manner:

(7) $$Q_{i}^{W}=W^{Q}A^{l-1}\;K_{i}^{W}=W^{K}A^{l-1}\;V_{i}^{W}=W^{V}A^{l-1}$$

where $A^{l-1}$ is the input to the lth hidden layer (l = 0, 1, 2). $W^{Q}$, $W^{K}$, and $W^{V}$ are network parameters. The score matrix in our proposed method is scaled by utilizing a Gaussian weighting matrix. This matrix is computed through the multiplication of key and query matrices, as described below:

(8) $$S_{i}=G_{S}^{l}\circ (\frac{Q_{i}^{\omega }(K_{i}^{\omega }{)}^{T}}{\sqrt{d}})$$

(9) $$V_{i}=G_{v}^{l}\circ V_{i}^{\omega }$$

(10) $$O_{i}=soft\max(S_{i})\circ V_{i}$$

$G_{s}^{l}$ is the Gaussian weight matrix.

Within the MSA block, a series of weight matrices in variables Q, K, and V are subjected to the same operating technique. This results in the calculation of multiple head-attention values. Afterwards, the outcomes of each individual head attention are combined. The mathematical representation of this process can be expressed by the following equation:

(11) $$MSA(Q,K,V)=Concat(O_{0},O_{1},...,O_{h-1})$$

where h represents the number of heads.

Ultimately, the characteristics acquired within the transformer are afterward inputted into the classifier for the purpose of categorization. This classifier comprises two fully linked layers, with the Sigmoid activation function being employed for the output of the final layer. The function maps the output values within the range of 0 and 1. Individuals with a value equal to or beyond a threshold of 0.5 are classified as engaging in electro-pilfering, whilst individuals falling below this threshold are categorized as regular users.

### 2.5. Overall Algorithm Steps

The overall process of the proposed DSDBGWT is shown in Algorithm 1.
**Algorithm 1** DSDBGWT Model**Input:** Input a dataset $X\in R^{1035\times 2}$; patch size s1 = 7; patch size s2 = 28; training sample rate = 80%.**Output:** Normal and abnormal prediction of test sets1: Set batch size to 100, optimizer Adam (learning rate: 10^−4^), epochs number e to 80.2: Perform patch1 in the X, available to $X\in R^{7\times 147\times 2}$ and divide them into training dataset and test dataset.3: Generate training loader and test loader.4: **for** i = 1 to e **do**5: Perform DSDB convolution layer.6: Perform patch 2 to change $X\in R^{7\times 147\times 16}$ $\mathrm{to}\;X\in R^{36\times 28\times 16}$.7: Perform a transformer network using Gaussian weighting.8: Spread the transformer output to pass into the classifier.9: Use the sigmoid function to identify the labels.10: **end for**11: Use test dataset with the trained model to get predicted labels.

## 3. Experimental Results and Analysis

### 3.1. Raw Electricity Consumption Dataset

The methodology was evaluated using a genuine dataset acquired from the State Grid Corporation of China. The dataset consists of a collection of daily power usage data series spanning from January 2014 to October 2016. This dataset encompasses a total of 43,272 customers. Approximately 8.55% of the aforementioned consumers were detected by the data source as participating in electricity theft operations and, as a result, were categorized as anomalous. We preprocessed the dataset according to the method in Section 2.1. It was randomly divided into five separate subsets of equal size while maintaining the original ratio of abnormal samples to normal samples. Four of these subsets were used as the training set to train the models, while the remaining subset was used as the test set to evaluate the models. The aforementioned procedure was iterated for the five potential choices, wherein a distinct subset was selected as the test set on each occasion. Consequently, five models are trained, with each model being evaluated on its respective test set to determine the test error. This process yields five test results, which are subsequently averaged. By repeating the aforementioned steps three times, the final results are obtained.

### 3.2. Experimental Setting

The experiments conducted in this paper were carried out on a server equipped with an Intel(R) Core (TM) i5-1035G1 CPU operating at a frequency of 1.7 GHz, with a maximum turbo frequency of 2.19 GHz. The server also had a total of 128 GB of RAM and was equipped with an NVIDIA GeForce RTX 3090 Ti GPU. The PyTorch 1.10.0 deep learning framework and Python 3.9 compiler were utilized on an Ubuntu machine to create the specific software. In the experiments, the batch size was set to 100, the learning rate was set to 0.001, the epoch was set to 80, and the Adam optimizer was used to make the model converge quickly.

### 3.3. Data Description

The chosen dataset is published by the State Grid of China and contains electricity consumption data of 43,272 electricity users over a period of 1035 days. The dataset contains electricity consumption data of 42,372 customers over a total of 1034 days from 1 January 2014 to 31 October 2016, of which 38,757 customers are normal electricity users (marked as 0) and the remaining 3615 customers are identified as electricity theft users (marked as 1). The details of the dataset are shown in Table 1.

**Figure 9:**
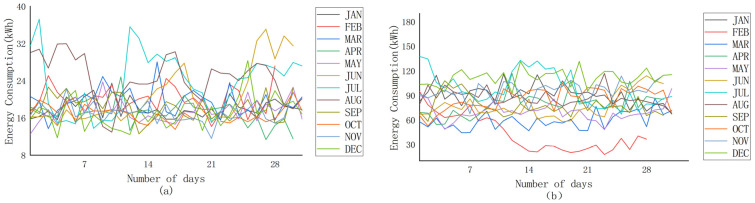
Average monthly electricity consumption in 2015. (**a**) Normal energy users. (**b**) Energy theft users.

**Figure 10:**
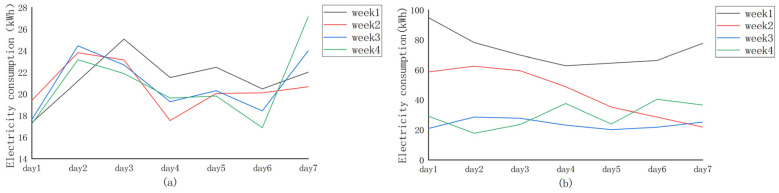
Average daily electricity consumption every four weeks (February 2015). (**a**) Normal energy users. (**b**) Energy theft users.

The anomalous manifestations of electricity theft are not only shown on the surface of the data, but their implied patterns and trends are equally characterized. In particular, Figure 9a gives an example of the electricity consumption data of a normal electricity user in one year (i.e., 2016), and Figure 9b represents an example of the electricity consumption data of an electricity theft user in one year. As can be seen from Figure 9, the electricity consumption data of normal users in July, August, and September are higher than that in other months (high air conditioning usage in summer), but overall are relatively stable. The overall data in other months are generally consistent with little fluctuation; the data of the electricity theft user appear to be abnormally chaotic, and the decline in electricity consumption in a certain month is particularly high, which is not in line with the normal pattern of electricity consumption. As shown in Figure 10, the electricity consumption data for four weeks (February 2015) of normal users and electricity theft users are extracted for further analysis. Figure 10a shows that, under normal circumstances, normal electricity users can exhibit significant periodicity, with weekly electricity consumption usually peaking on day 2 or 3, often reaching a low on day 4, and then starting to rise again, whereas the electricity consumption data for those defined as stealing (Figure 10b) fluctuates cyclically for the first two weeks (i.e., week 1 and week 2). However, from the second week onwards, electricity consumption decreases significantly and, thereafter, electricity consumption remains at a low level.

**Figure 11:**
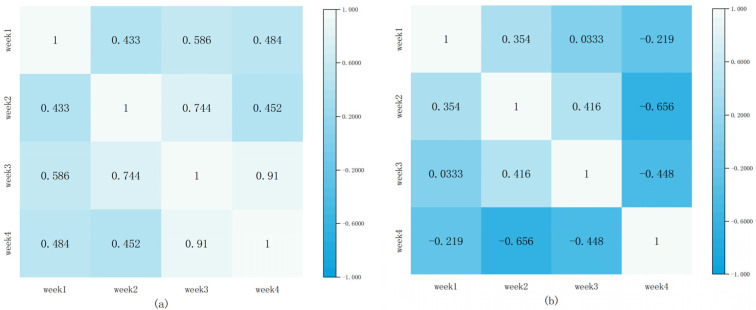
PCC of electricity consumption by week (February 2015). (**a**) Normal energy users. (**b**) Energy theft users.

In order to better analyze the periodicity of normal customers and the non-periodicity of electricity theft users, we performed a correlation analysis on the electricity consumption data. Figure 3 shows the Pearson correlation coefficient (PCC) of the electricity consumption of the above two users over a four-week period. In this case, Figure 11a shows the PCC values for normal users and Figure 11b shows the PCC values for electricity theft users. From Figure 11a, we can find that the electricity consumption data of normal users have a strong positive correlation. Most of their PCC values are around 0.5, and some even reach 0.9 (a closer PCC value to 1 means that a stronger correlation [30]), whereas the PCC value of the electricity consumption data of abnormal users is not more than 0.4 (Figure 11b), and even the phenomenon of negative PCC values occurs, which means that they show a negative correlation.

By statistically analyzing the electricity consumption data of normal users and electricity theft users, we can find that the electricity consumption data of electricity theft users are usually not periodic or non-periodic compared to normal users. Therefore, weekly, monthly, quarterly, and annual electricity consumption data can be used as benchmarks for feature extraction.

### 3.4. Evaluation Indicators

In order to evaluate the efficacy of the model, its performance was assessed using various metrics, including precision, recall, F1 score (F1), Area Under the Curve (AUC), and Mean Average Precision (MAP). The measurements encompass four primary error rates, namely, false positive (FP), false negative (FN), true positive (TP), and true negative (TN) [2,31]. 

The recall metric is defined as the ratio of accurately recognized instances of electricity theft by the model to the total number of real electricity theft samples:

(12) $$recall=\frac{TP}{TP+FN}$$

Precision is a metric that quantifies the proportion of samples accurately identified by the model as instances of power theft relative to the overall number of samples categorized as instances of electricity theft across all detection tests:

(13) $$precision=\frac{TP}{TP+FP}$$

The F1 score, also known as the balanced score, is a statistical measure used to assess the precision of a binary classification model. The evaluation metric takes into account both the precision and recall of the classification model:

(14) $$F1=\frac{2\times precision\times recall}{precision+recall}$$

AUC is defined as the area under the ROC curve and is used to measure the overall quality of the classifier. The larger the value of AUC, the better the performance of the classifier:

(15) $$AUC=\frac{\sum_{i\in positiveClass}Rank_{i}-\frac{M(1+M)}{2}}{M\times N}$$

where $Rank_{i}$ denotes the rank value of sample i, M is the number of normal samples, and N is the number of electricity theft samples.

MAP is a position sensitive indicator; if the abnormal samples are ranked higher than the normal samples, the higher the value of MAP. It can be calculated as follows:

(16) $$MAP@K=\frac{1}{m}\sum_{i=1}^{m}\frac{i}{p_{i}}$$

Considering the top K users in the sorted list, m is the number of selected users who have actually performed a power theft operation and $p_{i}(i=1,2,3,...,m)$ denotes the position of each anomaly in the sorted list. In our experiments, we compute this metric for all samples in a given list and abbreviate the metric as MAP@ALL.

### 3.5. Comparison with Advanced Methods

In order to demonstrate the efficacy of the suggested model, a selection of representative methodologies has been chosen to perform comparative tests using the DSDBGWT model. These methods integrate both representative and high-level scholarly publications with publicly accessible source code, spanning the period from 2001 to 2022. It is noteworthy to emphasize that the aforementioned methods were applied to a preprocessed dataset in order to ensure a fair comparison:

(1) Random forest (RF) [44]: The RF classifier, also known as random forest, is a machine learning algorithm composed of several decision trees;
(2) MiniRocket [45]: The MiniRocket model is a time series classification model that operates at rapid speeds. It utilizes a concise collection of predetermined convolutional kernels to convert the input time series data. The extracted features are subsequently employed in the training of a linear classifier;
(3) Wide and Deep CNN (Wide and Deep) [29]: The Wide and Deep model, which has a wide component and a deep CNN component, has gained significant traction as a fundamental approach in various domains;
(4) Hybrid-Order Representation Learning [40]: The electrical behavior classifier employs a comprehensive representation that combines first-order and second-order variables to detect occurrences of electricity theft;
(5) Hybrid Attention (HyAttn) [39]: The extraction of features is performed using a convolutional module that is enhanced by an MSA technique. Subsequently, the classification of these features is carried out evenly by concatenating convolutional layers with a kernel size of 1.

To ensure the integrity of the experimental findings, the network architecture and associated parameters from both classical and contemporary methodologies in the existing literature are employed to replicate the models for comparison studies. All tests were conducted using identical hardware configurations and maintained a consistent ratio of training to testing samples. The empirical findings are shown in Table 2.

Table 2 presents a comprehensive overview of the performance exhibited by all the approaches that were compared. The classification methods RF and MiniRocket, although known for their strong performance, are not specifically tailored for the purpose of power theft detection. The utilization of a Wide and Deep CNN in a CNN-based framework yields forecasts that are more dependable. The Wide and Deep CNN exhibits the capability to capture periodicity in weekly patterns through the utilization of deep CNN models and the integration of global knowledge from wide components. However, the performance of the model is constrained by the simplistic approach of stacking convolutional and fully connected layers, resulting in limited effectiveness for long-distance feature extraction and consequently leading to its poor accuracy. The HORLN model leverages first-order information to conduct shallow feature extraction on the sample sequence. Subsequently, the recovered features from the first-order information are employed as input for second-order processing. Despite the implementation of shallow feature extraction and long-distance feature extraction, the current model lacks the necessary level of granularity. HyAttn significantly enhances performance by integrating extended convolutional layers and including a self-attention mechanism. This approach effectively leverages both CNN and SA to extract shallow features and long-distance features from the input data simultaneously. However, it lacks selectivity in extracting features across long distances and does not dynamically adjust the weights of feature extraction across tokens while considering temporal considerations. The extracted features are not sufficiently complete, leaving potential for further improvement in accuracy. The model proposed in this article, known as the DSDBGWT model, incorporates a DSDB structure to enhance the extraction of comprehensive feature information during shallow feature extraction. Additionally, it incorporates Gaussian weighting processing on the token during training, enabling accurate and efficient feature extraction and F1 score calculation. The AUC and MAP@ALL metrics exhibit increases of 3.28%, 1.76%, and 0.36% compared to the highest values achieved by the aforementioned methods.

### 3.6. Parametric Analysis

The examination of parameters examines several elements that impact both the performance of classification and the process of training. The factors encompassed in this analysis consist of the number of output channels inside the convolutional network, the count of tokens, and the number of heads involved in the multi-head attention mechanism.

**Figure 12:**
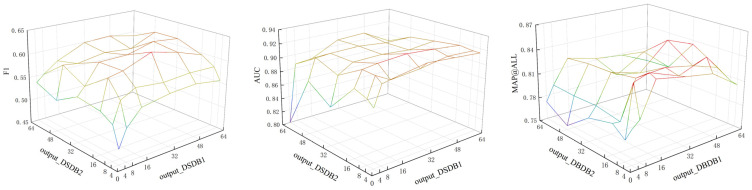
The impact of the number of output channels in CNN on various evaluation metrics.

The augmentation of channels within the convolutional kernel improves the model’s ability to extract features. However, this augmentation also introduces greater complexity to the model, which can potentially result in overfitting issues. The discussion revolves around the number of output channels in the two convolutional layers of the convolutional neural network. The impact of this parameter on F1, AUC, and MAP@ALL metrics is illustrated in Figure 12. The number of output channels for the first convolutional layer is denoted as output_DSDB1, whereas the number of output channels for the second convolutional layer is denoted as output_DSDB2. Based on the data presented in Figure 12, it can be observed that F1 achieves optimal performance when the values of output_DSDB1 and output_DSDB2 are set to 32 and 16, respectively, resulting in a performance metric of 0.629. Additionally, this configuration corresponds to the largest AUC value of 0.923. In the context of MAP@ALL, the maximum value is observed at output_DSDB1 = 48 and output_DSDB2 = 32, with a corresponding value of 0.848. In terms of the parameters, if the number of output channels of output_DSDB1 is doubled, it will lead to a doubling of the number of input channels of output_DSDB2. Consequently, this will not only increase the parameters of output_DSDB1, but will also increase the parameters of output_DSDB2. The excessive number of parameters can negatively impact the efficiency of the model. Therefore, in order to maintain model accuracy, measures need to be taken. Simultaneously, it is imperative to minimize the selection of output channels. After considering all relevant factors and analyzing the experimental findings, we have determined that the optimal number of output channels is output_DSDB1 = 32 and output_DSDB2 = 16. At this configuration, the corresponding values for F1, AUC, and MAP@ALL metrics are 0.629, 0.923, and 0.834, respectively.

The computational cost is directly influenced by the quantity of tokens in MSA. To regulate the CNN output features at various scales, we employ patching, which ultimately controls the quantity and dimensions of tokens. The fine-grained characteristics are influenced by the number of tokens, while the receptive field of the token features is determined by the dimension. The findings shown in Table 3 demonstrate the impact of token count on F1, AUC, and MAP@ALL within the context of MSA. The observed sample sequence exhibits periodicity not just on a weekly basis, but also on monthly and quarterly time scales. When the number of P is 7, 28, or 91, these correspond to the studies conducted in weekly, monthly, and quarterly patches, respectively. The table presents the performance metrics of F1, AUC, and MAP@ALL for different token values. It is seen that, when P = 28, F1 achieves a value of 0.629 and AUC achieves a value of 0.923. Comparatively, the impact of P = 7 and P = 28 on MAP@ALL is similar. Therefore, P = 28 (token = 36) is selected as the input for the transformer network. Based on the findings, it can be inferred that, while the transformer network exhibits strong capability in handling long-distance dependencies, its effectiveness is not only determined by the length of the sequence; rather, there exists a specific range within which the network performs optimally.

The primary purpose of employing multiple heads is to concurrently execute numerous independent attention computations, while also connecting their respective outputs. The use of multi-head attention in neural networks enhances the capacity to capture more comprehensive feature information. Similar to how raising the number of channels in a convolutional kernel in a CNN amplifies model complexity, augmenting the number of attention heads in multi-head attention similarly substantially elevates model complexity. The impact of the number of heads in the multi-head attention mechanism on each evaluation parameter is depicted in Table 4. Based on the data presented in the table, it is evident that the F1 score exhibits an upward trend as the number of heads increases, particularly when the number of heads is relatively small. Notably, the F1 score reaches its peak value of 0.629 when the number of heads reaches 48. However, a gradual decline in the F1 score is observed as the number of heads further increases to 64 and 80. This observation demonstrates that an excessive number of heads is not essential. When a sufficient number of heads are present, this enables comprehensive utilization of all aspects of the feature information. However, as the number of heads increases, so does the number of parameters and the computational load. Consequently, this leads to a decrease in the efficiency of the model. In conclusion, the value of h = 48 was selected as the designated quantity of heads for the MSA.

### 3.7. Ablation Experiments

**Figure 13:**
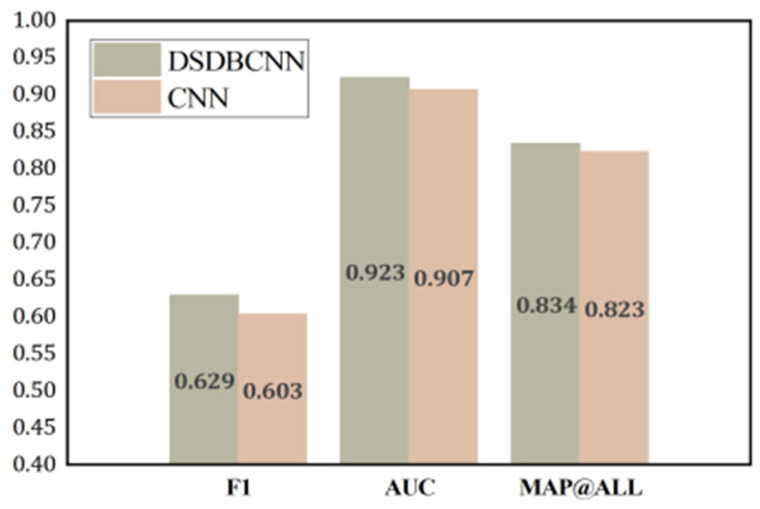
Performance comparison results between CNN with DSDB architecture and traditional CNN.

To assess the efficacy of the multi-branch component, we substitute it with a conventional two-dimensional convolution kernel for verification purposes. In this particular case, the substitution of a 1 × 3 and 3 × 1 convolution kernel is made with a 3 × 3 convolution kernel, while leaving other structures unaltered. The classification results obtained from the SGCC dataset are depicted in Figure 13. The figure demonstrates that the suggested model exhibits enhancements in the F1 score, AUC, and MAP@ALL by 4.31%, 1.76%, and 1.33%, correspondingly, in comparison to the model ordinary convolution. This is because the 1 × 3 convolution kernel in the dual-branch structure we designed efficiently extracts the intra-week features in the power data, and the 3 × 1 convolution kernel efficiently extracts the intra-week features in the power data. The experimental results show that the proposed dual-branching part can enhance the feature extraction ability of the network model and that the scheme is feasible.

The proposed model utilizes a fusion of DSDB CNN and GWT techniques for the purpose of identifying instances of electricity theft among customers. A series of ablation experiments were performed on the SGCC dataset to comprehensively evaluate the efficacy of the approach. These experiments involved testing various combinations of components. Table 5 examines five combinations and evaluates the influence of various components on the overall model in terms of classification accuracy. “√” indicates that the structure is added to the model, and “×” indicates that the structure is not used in the model.In this context, DSDB refers to a CNN with a DSDB structure. The term “conv” denotes the utilization of a regular 2D convolutional kernel. G.W. symbolizes the incorporation of Gaussian weighting treatment into the transformer model. Lastly, “tran” refers to the transformer network without Gaussianization. In Variant (1), the utilization of solely CNN is limited due to the absence of transformers. Consequently, the receptive field is restricted, leading to the extraction of primarily local information. As a result, the achieved F1 score is quite low. Variant (2) refers to the utilization of the tran network exclusively for long-distance feature extraction, while neglecting the use of CNN for shallow extraction of samples. Consequently, this approach exhibits limited capability in capturing local information and is susceptible to the issue of gradient vanishing. As a result, its F1 score is notably low, measuring only 0.426. Variant (3) entails the fusion of CNN with transformer. It is evident that the combination of these two models yields significantly improved accuracy compared to their individual implementations. This finding underscores the importance of incorporating both local and global temporal dependencies in the context of power theft detection. Notably, the F1 score of this combined approach reaches a value of 0.597. In (4), we use CNN with DSDB structure and transformer for combination. One can see that its accuracy is a little better than (3), which perfectly proves the effectiveness of the DSDB structure. In accordance with premise (3), we applied Gaussian weighting to the transformer, as described in (5). This approach considers both shallow and long-distance feature extraction, while also incorporating Gaussian weighting based on token distance closeness. As a result, the F1 score exhibits a 1% improvement compared to the approach outlined in (3). In (6), we once again integrate the CNN with DSDB structure using GWT. We replace the k × k convolution kernel with 1 × k and k × 1, allowing for simultaneous extraction of both inter-periodic and intra-periodic features. This modification not only enhances the model’s efficiency by reducing parameter usage, but also improves its accuracy. The F1 score exhibits a significant increase of 5.36% when compared to the value obtained in (3). In conclusion, the examination of the amalgamated experimental findings serves to reinforce the soundness and credibility of our theoretical framework.

## 4. Conclusions

This research presents a novel approach for power usage anomaly identification by proposing a hybrid network that combines a DSDB CNN with a GWT network. The proposed model incorporates a DSDB to perform shallow feature extraction on the sample sequence. This approach not only enables the extraction of more comprehensive features but also efficiently decreases parameter usage and enhances efficiency. The GWT network is capable of extracting characteristics from long-distance sequences in a more reasoned manner by utilizing the Gaussian-weighted technique. To assess the efficacy of the approach, a comparative experiment was undertaken, employing DSDBGWT alongside other classification methods. The experiment was performed on the publicly available dataset of SGCC. The experimental findings demonstrate that the approach described in this research study is capable of effectively extracting the abnormal characteristics of power consumption from the provided training samples. Moreover, the method exhibits a notable enhancement in F1 performance, surpassing the current state-of-the-art method by a margin of 3.28%. This improvement signifies a significant advancement over the existing advanced method. The technique described in this study is limited to feature extraction from data on electricity consumption. In actuality, a variety of complex factors, like the weather, holidays, the economy, etc., also influence how much power people use. The proposed DSDBGWT has good scalability in the high-level semantic feature extraction of multimodal data. In the future, we will build on the DSDBGWT model by fusing the model with more modes of data to extract high-level features of electricity consumption sequences, thus further improving the classification accuracy.

## Figures and Tables

**Table 1:** Raw data status.

Description	Value
Total number of electricity consumers	42,372
Number of abnormal electricity consumers	3615
Time span	1 January 2014–31 October 2016
Proportion of missing data	25.7%
Maximum daily consumption of electricity by customers	2782.2

**Table 2:** Performance comparison of different methods.

Methods	F1	AUC	MAP@ALL
RF [44]	0.386 ± 0.011	0.804 ± 0.018	0.603 ± 0.011
MiniRocket [45]	0.427 ± 0.008	0.829 ± 0.013	0.683 ± 0.009
Wide and Deep CNN [29]	0.468 ± 0.004	0.862 ± 0.011	0.751 ± 0.007
Hybrid-Order Representation Learning [40]	0.594 ± 0.004	0.895 ± 0.007	0.807 ± 0.006
HyAttn [39]	0.609 ± 0.003	0.907 ± 0.006	0.831 ± 0.006
DSDBGWT (proposed)	**0.629 ± 0.002**	**0.923 ± 0.004**	**0.834 ± 0.004**

**Table 3:** The impact of the number of tokens in transformer networks on various evaluation metrics.

P, Token	F1	AUC	MAP@ALL
P = 7, Token = 147	0.570	0.915	0.835
P = 28, Token = 36	0.629	0.923	0.834
P = 91, Token = 11	0.576	0.903	0.816

**Table 4:** The impact of the number of heads in multi-head attention mechanism on various evaluation metrics.

h	F1	AUC	MAP@ALL
16	0.616	0.922	0.813
32	0.618	0.917	0.833
48	0.629	0.923	0.834
64	0.620	0.921	0.827
80	0.612	0.919	0.816

**Table 5:** Performance of different variants of DSDBCGW.

	DSDB	conv	G.W.	tran	F1	AUC	MAP@ALL
(1)	×	√	×	×	0.562	0.874	0.827
(2)	×	×	×	√	0.426	0.830	0.737
(3)	×	√	×	√	0.597	0.909	0.816
(4)	√	×	×	√	0.599	0.897	0.815
(5)	×	√	√	√	0.603	0.907	0.823
(6)	√	×	√	√	0.629	0.923	0.834

## Data Availability

Not applicable.
